# A german-language competency-based multisource feedback instrument for residents: development and validity evidence

**DOI:** 10.1186/s12909-020-02259-2

**Published:** 2020-10-12

**Authors:** Eva K. Hennel, Ulrike Subotic, Christoph Berendonk, Daniel Stricker, Sigrid Harendza, Sören Huwendiek

**Affiliations:** 1 Department for Assessment and Evaluation (AAE), Institute for Medical Education, University of Bern, Mittelstrasse 43, 3012 Bern, Switzerland; 2 University Children’s Hospital Basel, Spitalstrasse 33, 4056 Basel, Switzerland; 3 Department of Internal Medicine, University Medical Centre Hamburg-Eppendorf, Martinistr. 52, 20246 Hamburg, Germany

**Keywords:** Multisource feedback, 360-degree, Workplace-based assessment, Assessment, Postgraduate training, Continuous professional development

## Abstract

**Background:**

In medical settings, multisource feedback (MSF) is a recognised method of formative assessment. It collects feedback on a doctor’s performance from several perspectives in the form of questionnaires. Yet, no validated MSF questionnaire has been publicly available in German. Thus, we aimed to develop a German MSF questionnaire based on the CanMEDS roles and to investigate the evidence of its validity.

**Methods:**

We developed a competency-based MSF questionnaire in German, informed by the literature and expert input. Four sources of validity evidence were investigated: (i) Content was examined based on MSF literature, blueprints of competency, and expert-team discussions. (ii) The response process was supported by analysis of a think-aloud study, narrative comments, “unable to comment” ratings and evaluation data. (iii) The internal structure was assessed by exploratory factor analysis, and inter-rater reliability by generalisability analysis. Data were collected during two runs of MSF, in which 47 residents were evaluated once (first run) or several times (second and third run) on 81 occasions of MSF. (iv) To investigate consequences, we analysed the residents’ learning goals and the progress as reported via MSF.

**Results:**

Our resulting MSF questionnaire (MSF-RG) consists of 15 items and one global rating, which are each rated on a scale and accompanied by a field for narrative comments and cover a construct of a physician’s competence. Additionally, there are five open questions for further suggestions. Investigation of validity evidence revealed that: (i) The expert group agreed that the content comprehensively addresses clinical competence; (ii) The response processes indicated that the questions are understood as intended and supported the acceptance and usability; (iii) For the second run, factor analysis showed a one-factor solution, a Cronbach’s alpha of 0.951 and an inter-rater reliability of 0.797 with 12 raters; (iv) There are indications that residents benefitted, considering their individual learning goals and based on their ratings reported via MSF itself.

**Conclusions:**

To support residency training with multisource feedback, we developed a German MSF questionnaire (MSF-RG), which is supported by four sources of validity evidence. This MSF questionnaire may be useful to implement MSF in residency training in German-speaking regions.

## Background

Feedback is one of the most important components of effective learning [1–3]. In particular, feedback can effectively support medical training by making individuals aware of gaps in knowledge or insufficient skills [4–6] and thus guide learning. Medical training should therefore include learning with the help of feedback, e. g. through workplace-based assessment.

There are several methods of workplace-based assessment: Mini-CEX [7] and DOPS [8] are two examples which focus on one occasion with a patient encounter or one skill rather than on the overarching performance. By contrast, multisource feedback (MSF) can cover competencies, e.g. from the CanMEDS framework [9], over a longer period of time and provides feedback from several perspectives [10, 11], resulting in meaningful feedback in the setting of competency-based training.

MSF can be used in a variety of settings, for a formative or summative purpose, during undergraduate and postgraduate training as well as for continuous professional development. For a better understanding of this study, we summarise the common features of MSF: Typically, MSF consists of feedback given to a trainee by several raters via structured questionnaires. Raters can come from the groups of peers, supervisors, medical and non-medical co-workers, and patients, and their written feedback is often transferred to and discussed in a conversation with the feedback recipient [6, 12–16]. MSF can promote medical training in the long term by providing regular feedback and supporting the formulation of individual learning goals [6, 11].

There are several, mainly English-language, instruments for implementing MSF, which are used for a broad variety of purposes [17–24]. These vary not only in terms of the physicians´ discipline and expertise but also with respect to the aim of the assessment. While in many settings, MSF is used for formative purposes, some regions have made it a mandatory part of the certification or re-certification process. Such instruments include mini-PAT for formative assessment during Foundation training in the UK [17], the Sheffield Peer Review Assessment Tool (SPRAT) for more senior physicians such as consultants and specialist registrars in paediatrics in the UK [18, 19], the Peer Assessment Review (PAR), which was initially used for family physicians in Canada [20] and later adapted for the certification of surgeons there [21], the Ottawa Clinic Assessment Tool for surgical residents working in outpatient clinics in Canada [22], the INCEPT for physician appraisal in the Netherlands [23], and the CEFP 360, which has been investigated for the process of revalidation for general practitioners in the UK [24].

It has been described that in order to clearly understand a questionnaire, it is important that it is administered in the respondent’s native language [25]. However, to date, there are no validated instruments for carrying out MSF for residents in the German language. Furthermore, from the internationally available instruments listed above, the mini-PAT comes closest to the contextual requirements in this study, but a mere translation of the mini-PAT does not fully meet these requirements for the following two main reasons: First, it was necessary to represent all CanMEDS roles, as the CanMEDS roles are official federal goals for residency training in Switzerland [26]. Second, we had to ensure that each item was formulated appropriately for residency training in Switzerland or Germany. To briefly explain the local context of residency training: In Switzerland, four formative assessments per year are recommended [27] and usually undertaken in the form of mini-CEX [11] or DOPS [11]; in Germany, one discussion on the training with a supervisor per year is mandatory [28]. To the best of our knowledge, most institutions limit themselves to these. As a result, MSF is rare in Switzerland and formative assessments like Mini-CEX and DOPS are rare in Germany. Taken together, the availability of an MSF instrument in the German language and aligned to the context might support the wider use of MSF and thus support residency training by fostering feedback provision. In the framework of the present study, we therefore developed a German-language MSF questionnaire and examined the evidence of its validity.

To assess the validity of an instrument, different sources of validity evidence can be sought [29–32]. For the purpose of the present study, we chose to investigate the criteria of validity proposed by Messick [31], as described by Cook and Beckman [29]. The latter authors define the following five sources of evidence: Content: Does the instrument represent the construct? Response process: Relation between intended construct and the thoughts of users; Internal structure: Reliability and factor structure; Relation to other variables which measure the same construct; and Consequences: What are the intended and unintended consequences of using this instrument?

In the present study, we explore four of these sources (content, response process, internal structure, and consequences) with respect to the developed German-language competency-based MSF instrument.

## Methods

### Context of our study

The instrument was developed and employed at the surgical clinic of the University Children’s Hospital Zurich. At this clinic, residents had been receiving mini-CEX and DOPS four times per year, but to date, have not received further training on structured feedback. As the director of residency training expressed a desire to improve residency training by implementing MSF, participation in MSF became a mandatory part of the training for all residents in the surgical clinic with the beginning of this study.

For implementation, we took into account the literature on MSF in medical training and followed the described best practice [13–16, 33–38] as far as possible and appropriate. This meant that all participants (residents, raters and supervisors) were trained at the beginning of the study. The training covered the objective of MSF at that department, the content and use of the MSF questionnaire with its ratings scale, and general rules on giving and receiving (written) feedback. Residents and raters who started their training later on, received information from the director of training and through a handout. Supervisors received additional training for the feedback conversation including the formulation of learning goals. In our study, for every resident, feedback was obtained from up to 15 raters, on average 12.5. Raters could be chosen by each resident from a pool of trained persons following a specific composition: four consultant paediatric surgeons, four residents, three nurses from the ward, two nurses from theatre, two consultant anaesthesiologists. We deliberately left out patients from the group of raters in this study as they would have needed another version of the questionnaire. Feedback was collected and anonymously transmitted to the resident within a structured feedback conversation led by a trained supervisor. The MSF questionnaire was administered as an online questionnaire using the online platform SurveyMonkey (surveymonkey.com). Furthermore, residents also completed a self-assessment on the same items included in the MSF questionnaire. In the feedback conversation, the results of this self-assessment were compared and contrasted with the feedback provided by the raters via the MSF questionnaires. This eventually provided the basis for the formulation of learning goals and next steps. For the numbers of participants and an overview of the procedure, see (Table 1).

**Table 1:** Participants of MSF

Number of run	Number of participants in the run	Number of ratings and raters	Group of raters
Run 1	47 residents	Up to 15 ratings per residents (on average 12.5). Exceptions in cases where not enough raters could be found because the work experience was too short.	The pool of trained raters contained 152 persons from different groups (e.g. consultants, nurses, peers). From this pool, every resident was supposed to choose up to 15 persons. For the 2nd and 3rd runs, these 15 could be chosen again or other persons could be chosen, depending on the contacts during work.
Run 2	32 residents. All 32 had taken part in run 1.	Again up to 15 ratings per resident.	
Run 3	2 residents. Both had taken part in runs 2 and 3.	Again up to 15 ratings per resident.	
	Sum of residents: 47	Sum of ratings: 1019	
Sum of MSFs	81 MSFs. Each MSF with on average 12.5 ratings sum up to 1019 ratings from raters. These were completed with 81 self-assessments (one per resident per run).		

In contrast to the actual participation in MSF, which was a mandatory part of the residents’ training, the participation in this validation study was voluntary. Participation in the MSF included giving feedback via the MSF questionnaire (raters) or discussing it in the feedback conversation (residents and supervisors). Participation in the study included first, the analysis of pseudonymised MSF questionnaires, self-assessments and pseudonymised documentation of the feedback conversation. Second, residents were invited to take part in an online survey for evaluation purposes. Third, from all three groups, some participants were invited to take part in a think-aloud study. All research data were pseudonymised or anonymised. The local committee of the Association of Swiss Ethics Committees on research involving humans deemed, based on the detailed study protocol, that no further approval was necessary. All participants provided written informed consent.

### Demographic data on the study participants

Residents who received MSF were pursuing a specialisation in either paediatric surgery (*n* = 32; 24 female, 8 male) or paediatrics (*n* = 15; 10 female, 5 male). The majority of the residents had between 1 and 5 years of work experience (*n* = 43), while the remainder had between 6 and 10 years of experience (*n* = 4). Raters, as far as their data are known, stem from a group of 31 consultant paediatric surgeons (13 female, 18 male; median work experience 14 years), 20 nurses from the ward (all female, median work experience 17 years), six nurses from theatre (5 female, 1 male, median work experience 21 years), 26 consultant anaesthesiologists (17 female, 9 male, median work experience 13 years), 46 residents from paediatric surgery (38 female, 8 male, median work experience 5 years), 17 residents from paediatrics (12 female, 5 male, median work experience 4 years), and six residents from other specialties (2 female, 4 male, median work experience 6 years). Data on raters cannot be fully provided for every rating, as by chance some of the pseudonyms overlapped.

### Development of the MSF questionnaire and investigation of validity

In line with Cook and Beckman [29], we took into account and investigated four sources of validity evidence: (i) content, (ii) response process, (iii) internal structure, and (iv) consequences.

#### Content

Content validity was targeted during the development of the instrument by a panel of experts and informing the instrument by respective frameworks and literature. This panel included the authors EH, US, CB, and SHu, who are physicians providing Swiss and German perspectives from the fields of internal medicine, surgery, paediatrics, transfusion medicine and medical education. The instrument should represent the chosen construct of a physician’s competence as adequately and completely as possible. Moreover, the instrument should be sufficiently universal for use not only for surgical training but also with regard to non-surgical medical competence. After reviewing the literature on existing MSF instruments [6, 12–15, 21, 33, 39, 40], we chose the mini-PAT questionnaire [17] as the basis for our MSF instrument as this was the closest to our affordances, including with respect to its competency-based nature. As the mini-PAT is based on the SPRAT, which itself is based on the UK’s GMP domains [19] and mapped against the UK Foundation curriculum [17], it did not completely mirror the CanMEDS roles. As the CanMEDS roles are official federal goals for residency training in Switzerland [26], it was decided that a mere translation would not be sufficient and that a questionnaire designed for the Swiss and German situation of residency training was necessary. In detail, the items of the mini-PAT were translated, discussed, and adapted by the expert group, with the Swiss and German residency training in mind. We discussed and revised all items in the expert group until consensus on the meaning and phrasing of each item was reached. Additionally, we integrated more aspects of all CanMEDS physician roles from the 2005 edition of the framework [9] into our MSF questionnaire. Item 10 “keeps an eye on patient safety”, item 14 “is open to feedback and learns from it” and item 15 “shows initiative and assumes responsibility” can be seen as complementing the CanMEDS roles Health Advocate, Scholar and Manager, respectively. We are aware, that the Health Advocate includes more aspects. But, as the contact with communities or speaking on behalf of populations is rarely a part of a resident’s work in most specialties, we did not include respective items. Furthermore, from the mini-PAT, we retained the two open questions on strengths and areas for improvement of the residents (questions 17 and 18), and the question on the integrity of the resident (question 21), as we viewed these to be important. To further emphasise the formative nature of MSF, we added two questions (questions 19 and 20) relating to working conditions and concrete suggestions for the improvement thereof. The resulting questionnaire is shown in Table 2. For the residents´ self-assessment, we used a questionnaire consisting of the very same questions but written from the first-person perspective.

**Table 2:** MSF questionnaire for residency training in German language (MSF-RG)

	Original item	English translation	CanMEDS role
*Items 1–16 are rated on a 5-point scale from “Unter meinen Erwartungen” (below my expectations), “Erfüllt meine Erwartungen grenzwertig” (marginally fulfils my expectations), “Erfüllt meine Erwartungen” (fulfils my expectations), “Über meinen Erwartungen “(above my expectations), to “Weit über meinen Erwartungen “(far above my expectations), and alternatively «Nicht beurteilbar» (unable to comment), with space provided for narrative comments directly after each item.*			
	Wie beurteilen Sie die Ärztin/den Arzt im Hinblick auf die folgenden Aspekte unter Berücksichtigung des Weiterbildungsstandes?	How do you assess the physician with regard to the following aspects, taking into account the level of training?	
	Die Ärztin/ der Arzt …	The doctor …	
1	… stellt korrekte Diagnosen.	... diagnoses patient problems correctly.	Medical Expert
2	… entwickelt angemessene Behandlungspläne.	… formulates appropriate management plans.	Medical Expert
3	… ist sich seiner eigenen Grenzen bewusst und bittet in der entsprechenden Situation um Hilfe.	… is aware of her/his own limitations and asks for help in that situation.	Medical Expert
4	… ordnet medizinische Maßnahmen im Bewusstsein der Kosten an.	… orders investigations in awareness of costs.	Manager
5	… hat ein gutes Zeitmanagement und setzt Prioritäten.	… manages time effectively and prioritises.	Manager
6	… verfügt über gute manuelle/technische Fähigkeiten.	… has good manual and technical skills.	Medical Expert
7	… führt die Krankengeschichte und Berichte zeitgerecht und präzise.	… keeps records in a timely and accurate manner.	Communicator
8	… kommuniziert adäquat mit Patienten und Angehörigen.	… communicates adequately with patients and family members.	Communicator
9	… bezieht psychosoziale Aspekte mit ein.	… involves psychosocial aspects.	Communicator
10	… behält die Patientensicherheit im Blick.	… keeps an eye on patient safety.	Health Advocate
11	… kommuniziert adäquat mit Kollegen.	… communicates adequately with colleagues.	Collaborator
12	… ist erreichbar und zuverlässig.	… is accessible and reliable.	Collaborator
13	… gibt gern Wissen an junge Kollegen weiter.	… likes to teach younger colleagues.	Scholar
14	… ist offen für Feedback und setzt es um.	… is open to feedback and implements it.	Scholar
15	… ist initiativ und übernimmt Verantwortung.	… shows initiative and assumes responsibility.	Manager
16	Wie bewerten Sie im Gesamteindruck diese Ärztin/ diesen Arzt?	How do you rate this doctor overall?	
*Questions 17 and 18 can be answered with narrative comments.*			
17	Was sind die besonderen Stärken der Ärztin/des Arztes?	What are the individual strengths of this doctor?	
18	In welchen Bereichen sollte die Ärztin/der Arzt sich insbesondere noch verbessern?	In which areas do you see a need for improvement?	
*Questions 19–21 can be answered with yes or no, and ask for narrative comments in the case of a “yes”.*			
19	Sehen Sie äußere Einflüsse, die die Leistung der Ärztin/des Arztes beeinträchtigen oder befördern?	Are there hindering or facilitating influences on this doctor’s work?	
20	Haben Sie Vorschläge zur Veränderung der Arbeitsbedingungen der Ärztin/des Arztes?	Can you suggest changes in this doctor’s working conditions?	
21	Haben Sie irgendwelche Zweifel an der Integrität oder Gesundheit der Ärztin/ des Arztes? Falls ja, nennen Sie Ihre Bedenken:	Do you have any doubts about this doctor’s probity or health? If yes, please state your concerns:	Professional

In formulating the questions, we drew on principles pertaining to the design and formulation of questionnaires [41]. In particular, we endeavoured to use phrasing that was as clear as possible. In accordance with the recommendation that items should describe the desired behaviour [15], all items of our MSF questionnaire are phrased with a positive orientation rather than neutrally. The expert group also adjusted the scale based on experience with other instruments. We chose to use a 5-point scale on which the borderline rating is not in the middle, but is rather second from bottom. In this way, “positive” ratings might be more widely distributed, meaning across three points of the 5-point scale. The rating is not compared to an absolute goal, as in the mini-PAT (“expectations for F2”), but is rather relative to the resident’s state of training (“taking into account the level of training”). For each item, in addition to the given scale, space for narrative comments was provided with the intention of encouraging concrete observations and suggestions for improvement [35]. The same scale (“taking into account the level of training”) and space for narrative comments were used in the self-assessment questionnaire.

#### Response process

All participants (raters, residents and supervisors) were trained for their respective task as described above. The technical implementation was made as simple as possible. According to Cook [29], data security and the way in which data are further used also contribute to the response process validity. We therefore took these factors into account and provided information thereupon as part of the participants’ training.

Before the MSF questionnaire was finalised, the comprehensibility and clarity of the items were examined in a think-aloud study with residents and raters. For this purpose, the instruments (MSF questionnaire and self-assessment questionnaire) were completed by eight persons (two representing the group of residents, six representing the group of raters) and then discussed in order to optimise the intended understanding. These insights led to some modifications of the instruments, resulting in the final versions of the two questionnaires. The participants of the think-aloud study were not excluded from the use of MSF or from the data of this study.

All narrative comments, which were invited for each item of the MSF questionnaire, were analysed to check whether the respective item was understood as intended. We also analysed “unable to comment” ratings, and related either the resident’s specialty or the rater’s occupation to the percentage of items answered.

For evaluation purposes, first, one question on the usage of the MSF questionnaire was posed directly at the end of the questionnaire (in both the MSF and the self-assessment questionnaire), asking whether any changes were necessary regarding technical aspects or content of the MSF questionnaire. Second, all residents were sent a short pseudonymised online survey for evaluation of the MSF, which asked about hindering or facilitating factors regarding the feedback conversation and supervisor, raters, and general conditions for MSF.

#### Internal structure

The instrument was employed to evaluate 47 residents on 81 occasions of MSF. Thirty-two residents received MSF twice (in run 1 and run 2) and two residents received MSF three times (in runs 1, 2 and 3). An exploratory factor analysis and a reliability analysis were performed for the first and second run of MSF. Here, the first run is defined as the sum of MSF ratings given to residents who received their first MSF, and the second run analogously.

When using MSF questionnaires, not all participants are expected to answer all questions, but can rather answer “unable to comment” when applicable [15]. Thus, the dataset of MSF typically has many missing values. Based on the content of items, these missing values are systematically rather than randomly distributed (e.g. are related to the raters´ occupations). We therefore decided not to impute or replace missing data. To use the maximum of available data, the correlation matrices, in which missing values were omitted pairwise for each correlation, were used as the basis for the factor analyses.

Generalisability studies were conducted to investigate the inter-rater reliability for the scale score. The analyses were conducted for the total score at the raters’ level for the 47 residents in run 1 and 32 residents in run 2, respectively. The generalisability studies show how many raters are needed per resident to achieve a reliable indication of a resident’s competencies [42]. Therefore, the total variance of the total score was decomposed into components associated with residents (c) and raters (r) nested (:) within residents and crossed (×) with the score (s), whereas residents served as the object of measurement. This (r:c) x s design allows for variance component estimation of two sources: (i) differences between residents (object of measurement) and (ii) differences between raters nested within residents´ judgements [43, 44]. Reliability indices (generalisability (G) coefficient) and standard error of measurement (SEM) are reported as a function of the number of raters per resident. For comparison purposes, the generalisability study was also calculated with the overall item 16 instead of the total score.

#### Consequences

Repeated counselling by the same supervisor and documentation of the agreed learning goals should ensure that individual support of the resident takes place. Beginning with the second MSF per resident, the ratings and learning goals from the previous MSFs could be looked up again in the feedback conversation. The follow-up on the learning goals depended on the resident and supervisor. We analysed the MSF-induced consequences by taking into account supervisors´ written documentation of all feedback conversations including the number of learning goals and their formulation (SMART or otherwise).

In order to investigate the progress of those 32 residents who participated in both runs, the mean scores they achieved were compared between the two runs using a dependent samples t-test. As the rating scale was “taking into account the level of training”, residents who (in real-life) developed their competencies further during their training remained at their particular rating (on the scale). Thus, the scores were not expected to differ between the runs.

The continuous opportunity to provide feedback on the instrument and process should ensure that unexpected consequences such as problems in terms of acceptance, usability of the MSF questionnaire or problems in the MSF process could be identified early on. We checked all available data (MSF questionnaire and questionnaire for self-assessment) for hints of unexpected consequences.

Statistical analyses were performed with SPSS for Windows version 25, the statistical computing language R [45], and variance components for generalisability analysis were calculated using G_String A Windows Wrapper for urGENOVA [46].

## Results

### Resulting MSF questionnaire

The resulting competency-based MSF questionnaire, shown in Table 2, consists of 15 questions and one global assessment of medical competence as well as five additional open questions. All original items of the MSF-RG are presented in their original order. English translations and mapping against CanMEDS roles are added for the purpose of this publication only and are not part of the MSF questionnaire. The MSF questionnaire is used digitally and introduced with the following sentence: “This questionnaire is supposed to reveal the personal strengths and weaknesses of a physician in order to achieve the best individual training.” („Dieser Fragebogen dient dazu, die persönlichen Stärken und Schwächen einer Ärztin/eines Arztes aufzuzeigen, um die bestmögliche individuelle Weiterbildung zu erreichen.“). The self-assessment questionnaire consists of the same questions, grammatically adjusted to the first-person perspective.

### Investigation of validity evidence

#### Content

We consider the validity of the content to be grounded in the development process of the MSF questionnaire, in which an interdisciplinary group of experts from several medical specialties and from the field of medical education defined the desired construct of medical competence on the basis of the CanMEDS roles. The mini-PAT [17] was taken as the basis and extended such that the resulting items cover all CanMEDS roles. The expert group formulated the items of the MSF questionnaire on the basis of the literature on MSF according to the formative objective of this MSF.

#### Response process

**Fig. 1:**
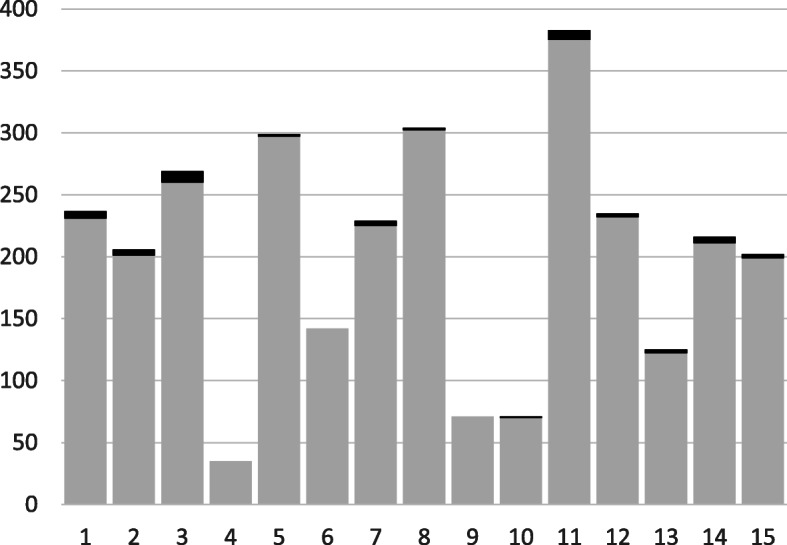
Analysis of narrative comments. Absolute number of answers which focus on the item as intended (grey) and the number of answers which focus on another topic (black) as a function of the items 1–15

The think-aloud study showed that with small adjustments, the items of the MSF questionnaire and self-assessment questionnaire were understood as intended. Analysis of all narrative comments for each item showed that the items were understood as intended in 96–100% of cases (see Fig. 1). The highest percentage of narrative comments which did not focus on the item as intended was seen for item 3 “… is aware of her/his own limitations and asks for help in that situation.”, which was also used to comment on more general aspects of collaboration and to comment on the residents´ limitations.

The analysis of “unable to comment” ratings is shown in Table 3. This analysis supports the response process and correct usage of the instrument by demonstrating that raters ticked “unable to comment” for items where valid judgments could not be given by the respective rater. To give some examples: Item 1 “... diagnoses patient problems correctly” was rated by many peers and consultants and some nurses from the ward, but rarely by persons from theatre. Item 8 “… communicates adequately with patients and family members” was rarely rated by nurses in theatre but often rated by nurses from the ward.

**Table 3:**
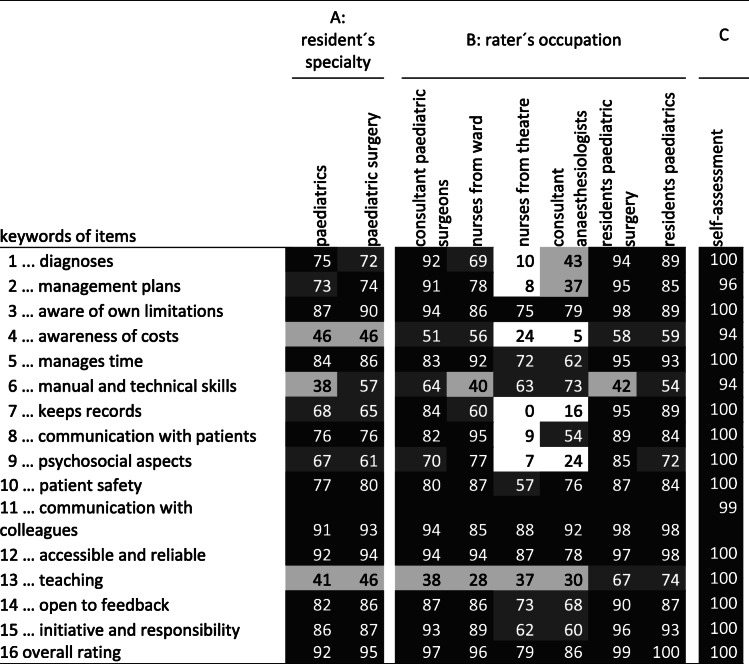
Analysis of “unable to comment” ratings as an aspect of the response process

Percentage of scale-based ratings of - column A: resident’s specialty, B: rater’s occupation, or C: self-assessment – each as a function of the items 1–16. Brightness of boxes emphasises the percentage of ratings: black: answered by 75% of raters or more, dark grey: 50–75%, light grey: 25–50%, white: less than 25%. Example: item 2 was answered in 73% of MSF for paediatric residents and in 8% of cases by nurses from theatre

Data from accompanying evaluations during the usage of the instrument confirmed the clarity of items and good usability: Raters made suggestions for changes to technical aspects or content of the MSF questionnaire in 62 cases from all 1019 ratings (6%). Residents made suggestions in nine cases from 81 self-assessments (11%). Raters mostly commented on the reasons why they chose “unable to comment”: Residents mostly commented on difficulties in matching their self-rating with the scale which reached from “below my expectations” to “far above my expectations”.

The more detailed online survey for evaluation from the residents’ perspective, regarding facilitating and hindering factors, indicated that the experienced MSF is feasible overall and well accepted without the need for major alterations. In terms of the feedback conversation, raters and general conditions, many facilitating factors were reported, which indicate the feasibility and high acceptance of MSF. Hindering factors mostly concerned discussions about the optimum time for MSF to take place during training. This means that some residents reported in the online survey that shortly after the beginning of a rotation (e.g. 8 weeks), it would have been too early to collect feedback, and conversely that MSF too close to the end of a rotation hindered them from effectively changing their behaviour.

#### Internal structure

##### Factor analysis

The KMO test showed that our data are suitable for factor analysis (KMO = 0.931 (first run), KMO = 0.921 (second run), Bartlett test < 0.001). An exploratory factor analysis was performed separately for the runs using the Kaiser criterion (drop all components with eigenvalues less than 1.0). This resulted in a single-factor structure for both runs. The one-factor solution accounted for 53.5% of the total variance in the first run and 62% in the second run. For an overview, the initial eigenvalues and the percentages of explained variance for the first three factors are listed in Table 4 for both runs.

**Table 4:** Eigenvalues and percentage of explained variance

	Factor	Eigenvalue	% of Variance	Cumulative %
Run 1	1	8.022	53.478	53.478
	2	0.912	6.077	59.556
	3	0.825	5.498	65.054
Run 2	1	9.309	62.062	62.062
	2	0.861	5.741	67.803
	3	0.689	4.591	72.394

Due to the skewed distribution of the answers in the individual items, it is to be expected that Cronbach’s alpha tends to underestimate the degree of internal consistency [47]. For this reason, we report two alternative measures in addition to Cronbach’s alpha, with omega total and the greatest lower bound (glb). The results are summarized in Table 5 for both runs. The 95% confidence interval is displayed for Cronbach’s alpha and omega total.

**Table 5:** Reliability measures and 95% confidence intervals

			95% confidence interval	
		Value	lower bound	upper bound
Run 1	Cronbach’s alpha	0.933	0.908	0.954
	omega total	0.946	0.925	0.962
	glb	0.962	–	–
Run 2	Cronbach’s alpha	0.951	0.932	0.967
	omega total	0.962	0.947	0.974
	glb	0.976	–	–

##### Inter-rater reliability

Table 6 depicts the variance components obtained by generalisability study (G-study). The obtained inter-rater reliability on the total score was 0.607 in the first run and 0.797 in the second run with a mean of 11.85 and 12.03 raters per resident, respectively. Compared to the total score, a slightly higher inter-rater reliability was found for the overall item 16, at 0.632 in the first and 0.827 in the second run, with a mean of 11.12 and 11.76 raters per resident, respectively. The results of the D-study are also presented in Table 6. A minimum of 12 ratings are needed in order to achieve an inter-rater reliability of 0.8 and seven ratings are sufficient for an inter-rater reliability of 0.7 in the second run. The also presented standard error of measurement (SEM) can be used to calculate the confidence intervals of the residents’ scores. Even with the G coefficient being smaller when compared with the overall item 16, the SEM is lower in all cases for the total score. This is due to the lower variance observed in the total score compared to the overall item 16.

**Table 6:** G-study and D-study on inter-rater reliability

		Estimated variance components				
		Inter-resident variance	Rater variance within resident	N	G-coefficient	SEM
Run 1	G-study	0.032 (0.077)	0.021 (0.045)	11.85 (11.12)	0.60 (0.63)	0.38 (0.46)
Run 2	G-study	0.093 (0.189)	0.024 (0.040)	12.03 (11.76)	0.80 (0.83)	0.28 (0.34)
	D-study	0.093 (0.189)	0.026 (0.042)	11	0.78 (0.82)	0.29 (0.34)
			0.028 (0.047)	10	0.77 (0.80)	0.30 (0.36)
			0.032 (0.052)	9	0.75 (0.79)	0.31 (0.37)
			0.035 (0.058)	8	0.72 (0.77)	0.32 (0.39)
			0.041 (0.066)	7	0.70 (0.74)	0.34 (0.41)
			0.047 (0.078)	6	0.66 (0.71)	0.36 (0.43)

Estimated variance components for the variance associated with residents and raters and the generalisability coefficient (G-coefficient) and standard error of measurement (SEM) for the first and second run (G-study). G-coefficient and SEM as a function of the number of rater ratings (N) for the second run (D-study). Results are presented for the total score and in parentheses for the overall item 16

#### Consequences

For all residents who expected another turn of MSF, between one and four tailored learning goals were written down, enabling residents to be individually guided during their training. In assessing the learning goals against the SMART formulation rules, most goals seemed to be specific, attainable, and relevant. However, hardly any goals were measurable or time-bound.

In order to analyse the progress between the first and second run, we compared the mean scores achieved per individual resident. The comparison revealed no significant difference (mean in run 1 = 3.34, mean in run 2 = 3.41; t _(31)_ = − 1.611, *p* = 0.117, η^2^ = 0.07), which indicates that residents developed as expected, as raters were asked to “take into account the level of training”.

**Fig. 2:**
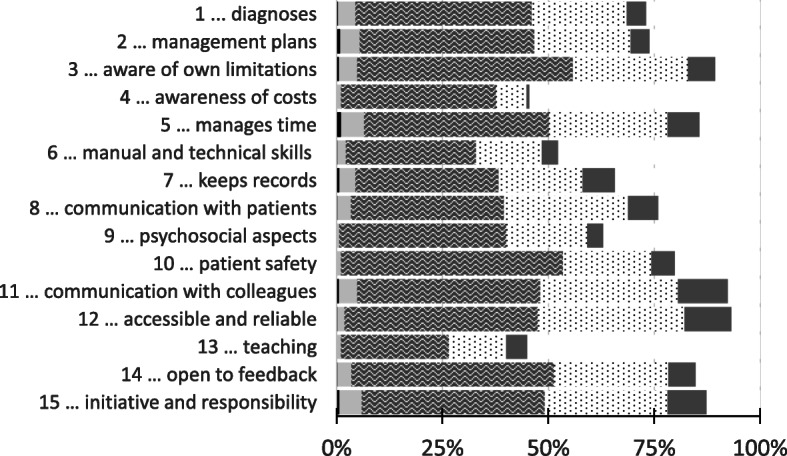
Answer patterns as a function of the items 1 to 16. For each item 1–16, the figure shows the relative amount of ratings per each point of the 5-point scale. Legend: from left to right: black: below my expectations, light grey: fulfils my expectations marginally, waved: fulfils my expectations, dotted: above my expectations, medium grey: far above my expectations. Missing to 100% are “unable to comment” ratings

To provide an overview, ratings as a function of the items are shown in Fig. 2.

No undesired consequences such as problems in terms of acceptance, usability of the MSF questionnaire, problems in the MSF process, or worsening of performance occurred.

## Discussion

We developed a German-language MSF questionnaire and investigated four sources of validity evidence following a model of proposed standards [29]. Our resulting MSF questionnaire (MSF-RG) consists of 15 questions and one global rating, which cover a construct of a physician’s competence, and five additional open questions for further suggestions for improvement. We found evidence for the validity of this MSF questionnaire regarding content, response processes, internal structure, and consequences.

*(i) Content validity* was supported by the deliberate development of the MSF questionnaire involving experts and basing the instrument on existing literature and the CanMEDS framework. When building the MSF questionnaire, its transferability to training in other, non-surgical specialties was also taken into consideration. In our setting, participants included residents in paediatric surgery as well as residents in paediatrics. The use of CanMEDS roles as a basis, the generic formulation of the items, and the interdisciplinary composition of the expert group should ensure that the instrument can be used in residency training in various specialist areas. At least in our setting with residents from paediatric surgery and paediatrics, who stem from the same hospital and worked in the same department during the time of this study, the implementation of the questionnaire was successful. This idea is in line with research by van der Meulen et al. and Mackillop et al. [23, 48], who found that one generic MSF can be used for various specialties, under the condition that the number of raters and their mix, as well as the content of items, is suitable for the respective purpose. Nevertheless, further studies are advisable to determine whether this is indeed also true for other contexts.

*(ii) Response process validity* was supported by the think-aloud study, as well as the analysis of narrative comments and analysis of “unable to comment” ratings, which indicate that overall, the focus of every item was understood as intended and that the instrument could be used as expected. Furthermore, comparison of data from the response process for the two groups of residents (paediatric surgeons compared to paediatricians) shows that the percentage of items answered on the scale is similar for both groups. Again, this finding, in line with the literature [23, 48], supports the notion that this generic MSF questionnaire can be utilised for both groups of residents. Data from evaluations confirmed the good usability and acceptance of the questionnaire.

*(iii) Internal structure*: The exploratory factor analysis indicated that a one-factor model fits the data and accounted for 53.5% of total variance in the first run and 62% of total variance in the second run.

The one-factor model might suggest that the scale does not differentiate as well as expected or that competence cannot be defined as the sum of separated skills but rather as their combination [49]. All indicators for internal consistency (Cronbach’s alpha, omega total and greatest lower bound (glb) values, and the generalisability studies when using the total score and the overall item 16) point to a redundancy of items. This finding is in line with a review by Wood et al. [15], who outlined several cases of multi-rater assessment for which they identified an “overwhelming halo effect” and summarised that often, an “interpersonal” factor would be the main influence on variety. Moreover, the authors mentioned that the need to divide performance into smaller semantic items nevertheless existed. We have a similar impression and would like to discuss this need in greater detail based on our findings. Despite the statistical results, the use of all items of the questionnaire makes sense for the following four reasons:

First, each item reflects a certain task or skill, which could perhaps be captured in an overarching factor, but without mentioning the task, it might be unlikely to receive a narrative comment. We think that especially CanMEDS roles which come to mind less directly, such as the Scholar or the Manager, might otherwise be forgotten. In this way, the variety of items helps to show the roles, which all are meant to be considered for the feedback.

Second, we have observed that for the formative purpose of the instrument, narrative comments are of much greater importance than scale-based ratings, in line with the literature [15, 35]. With items which mirror the desired behaviour and focus on specific tasks, it is easier to gain specific task-focused feedback instead of general comments. Thus, the pre-formulated items probably help to obtain a high quality of feedback in the narrative comments.

Third, supervisors and residents reported (data not shown) that the ratings for each scale served as a kind of “screening” parameter to obtain a quick overview of the current performance, compare easily between runs and facilitate the comparison of self-assessment with MSF ratings.

Fourth, the content validity evidence and the vast response process validity evidence gained within this study (see above) support the use of the reported scale with all the items.

Taken together, despite the statistical finding of a one-factor model, we advise to include all the items of the instrument with both the quantitative scale and the narrative comments to allow its full potential with regard to its formative purpose.

With respect to the higher inter-rater reliability (G-study) of .797 for the second run compared to .604 for the first run, we conclude that experience with the instrument is important to achieve an acceptable level of inter-rater reliability. This finding was similarly described by Moonen-van Loon et al. [50], who reached higher reliabilities by combining the results of several occasions of MSF over a prolonged period of time. The D-study showed that a minimum of 12 ratings were needed in order to achieve an inter-rater reliability of 0.8 and seven ratings were sufficient for an inter-rater reliability of 0.7 in the second run.

The finding that the scores are valid and reliable implies that data collected with 12 raters, composed as described, can be used to rate a doctor’s competency in order to provide formative feedback based on this questionnaire. This number of raters is consistent with previous literature reporting that between five and 20 raters are necessary for an accurate rating, depending on raters´ occupations, on the roles assessed and on the training of raters [15]. With the formative use of the instrument in mind, fewer raters could theoretically be taken into consideration for the second run. However, fewer raters might reduce the number of narrative comments and the variety of perspectives.

*(iv) The consequences* of our instrument were demonstrated by the learning goals which were formulated and by the investigation of progress between the first and second run of MSF. Regarding the learning goals: Though supervisors had been trained on how learning goals should be formulated, including SMART goals, the resulting learning goals fulfilled only some aspects of SMART. Training not only the supervisors but also the residents in this respect, might make a difference. Regarding the scale-based ratings as compared between the two runs, we did not find a significant difference, which indicates that residents developed their competencies further as expected, as raters were asked to “take into account the level of training”. This result of the t-test can be caused by various reasons. We expect that it is attributable to the growth of residents due to their training in combination with MSF. Other reasons might be growth due to residency training independently of MSF, or maturation during the study time, independently of training or MSF. Other specific influences cannot be ruled out, but to the best of our knowledge are unlikely. Though more data on consequences are needed, our results contribute to the knowledge on MSF, as the consequential validity of MSF instruments is rarely reported in the literature [16, 37, 50]. Further investigation of this aspect of validity is planned by including more participants and investigating long-term consequences.

The main strength of our study is that, to the best of our knowledge, MSF-RG is the first competency-based MSF instrument in the German language for which different sources of validity evidence have been investigated. As we chose to draw on the widely used CanMEDS framework and piloted our MSF instrument for surgical as well as non-surgical residents, we assume that its use should be transferable to other specialties.

A further strength of the study is that we described not only the internal structure but also the response process and consequences in detail, thus addressing a gap in the literature. A recent review on the validity of MSF summarized that these aspects were reported too seldom, and as a result, knowledge on these was still limited [51].

The results of our study are limited by the fact that all participants, although having different training goals (paediatric surgery, paediatrics), worked in one department, which reduces generalisability. Additional research on the validity of the instrument is needed and will be feasible as soon as more data with more participants from diverse institutions have been collected.

Further, the results of the t-test, which was used to investigate the progress of residents between run 1 and run 2 of MSF, have to be interpreted with caution as we can only guess that the use of MSF is one of the factors facilitating the residents´ progress.

It was not possible to investigate the “relation to other variables”, which is the fifth source of validity evidence according to Cook et al. [29], as this institution does not currently use any other measurement which also rates the complete construct of a physician’s competency at several time points during training.

Moreover, the perspective of patients and their parents was not included in this study. As their view on a physician’s competence is highly important to gain a more comprehensive picture, we propose that an additional questionnaire for this group of raters should be developed. Then, it should also be investigated whether this perspective might influence the process of MSF and its validity evidence.

Future research should look more closely at the consequences of the MSF for the learning goals of residents and its impact on the performance of all participants, which could help to further foster the understanding of MSF.

Since the validity of an instrument depends on its use, we recommend that the extensive literature on the best possible implementation of MSF should be considered [13–16, 33–38] when planning to implement MSF.

## Conclusion

We developed a German-language competency-based questionnaire for the implementation of multisource feedback in postgraduate medical training, and examined four sources of validity evidence with 47 residents on 81 occasions of MSF. We believe that our study could also serve as an example for others on how to develop and validate an instrument which is primarily based on an existing international instrument to account for both content adaptations and native language of users. We found evidence for the validity of this MSF questionnaire regarding content, response processes, internal structure and consequences. This suggests that this MSF questionnaire in German (MSF-RG) is suitable for MSF to support residency training. Further studies will be needed to investigate the long-term consequences of the instrument as well as the correlation with scores from other assessments.

## Data Availability

The datasets generated and analysed during the current study are not publicly available as this might endanger the anonymity of participants, but are available from the corresponding author upon reasonable request.

## Abbreviation

MSF: Multisource Feedback
